# The Chemical Treatment of Tuberculosis

**Published:** 1916-04-01

**Authors:** 


### THE CHEMICAL TREATMENT OF TUBERCULOSIS.

During the last three years the treatment of tuberculosis in this country has moved along well-recognised and definite lines; a broad and comprehensive view of the whole problem has been taken, and the opinion is now definitely held that the end to be aimed at is the gradual control and final abolition of the disease rather than efforts at securing brilliant clinical results in individual cases. While health authorities are working systematically and persistently in their efforts to control the ravages of the tubercle bacillus the investigator in the laboratory is also untiring in his efforts to discover a specific remedy for the disease. The latest in the field is Professor Renon, who is covering old ground in his endeavour to find some chemical substance which will check the virulence and growth of the tubercle bacillus without causing injury to the organism. Professor Renon's view that the cure of tuberculosis can only be secured by chemical treatment, and not by means of vaccine or serum-therapy, brings to mind a host of chemical substances which in the past have been recommended, and more or less extensively tried in the treatment of tuberculosis. One would have thought that the day of the chemical treatment of tuberculosis had gone never to return, but the results obtained by the treatment of syphilis with salvarsan have no doubt revived investigation into the action of chemical substances in inhibiting the growth of the tubercle bacillus. Professor Renon has been experimenting with silver, gold, mercury, selenium, and other salts, but it is to be regretted that particulars of his experiments should have appeared at this early stage in the public Press, as the result is but to accentuate the "spes Phthisica" of the advanced and incurable consumptive. While every encouragement must be given to experimental investigation on the treatment of tuberculosis, it is to the war of attrition against the tubercle bacillus at present carried on by health authorities that we must look for final success. An illuminating light on the possible future of tuberculosis is shed by the history of leprosy. Long before the causative agent of that disease was discovered it had ceased to exist in this country as a result of the stringent precautionary measures which had been adopted. And it is by no means improbable that tuberculosis as a disease of the masses in this country will have ceased to exist long before a specific cure has been discovered.

